# Food purchase behaviour in a Finnish population: patterns, carbon footprints and expenditures

**DOI:** 10.1017/S1368980022001707

**Published:** 2022-11

**Authors:** Jelena Meinilä, Hanna Hartikainen, Hanna L Tuomisto, Liisa Uusitalo, Henna Vepsäläinen, Merja Saarinen, Satu Kinnunen, Elviira Lehto, Hannu Saarijärvi, Juha-Matti Katajajuuri, Maijaliisa Erkkola, Jaakko Nevalainen, Mikael Fogelholm

**Affiliations:** 1 Department of Food and Nutrition, University of Helsinki, PO Box 66, Helsinki 00014, Finland; 2 Tampere University, Tampere, Finland; 3 Natural Resources Institute Finland, Helsinki, Finland; 4 Department of Agricultural Sciences, University of Helsinki, Helsinki, Finland; 5 Helsinki Institute of Sustainability Science, University of Helsinki, Helsinki, Finland; 6 Department of Teacher Education, University of Helsinki, Helsinki, Finland

**Keywords:** Nutrition, Greenhouse gas emission, Diet, Food consumption, Environmental impact

## Abstract

**Objective:**

To identify food purchase patterns and to assess their carbon footprint and expenditure.

**Design:**

Cross-sectional.

**Setting:**

Purchase patterns were identified by factor analysis from the annual purchases of 3435 product groups. The associations between purchase patterns and the total purchases’ carbon footprints (based on life-cycle assessment) and expenditure were analysed using linear regression and adjusted for nutritional energy content of the purchases.

**Participants:**

Loyalty card holders (*n* 22 860) of the largest food retailer in Finland.

**Results:**

Eight patterns explained 55 % of the variation in food purchases. The *Animal-based* pattern made the greatest contribution to the annual carbon footprint, followed by the *Easy-cooking*, and *Ready-to-eat* patterns. *High-energy*, *Traditional* and *Plant-based* patterns made the smallest contribution to the carbon footprint of the purchases. *Animal-based*, *Ready-to-eat*, *Plant-based* and *High-energy* patterns made the greatest contribution, whereas the *Traditional* and *Easy-cooking* patterns made the smallest contribution to food expenditure. Carbon footprint per euros spent increased with stronger adherence to the *Traditional*, *Animal-based* and *Easy-cooking* patterns.

**Conclusions:**

The *Animal-based*, *Ready-to-eat* and *High-energy* patterns were associated with relatively high expenditure on food, suggesting no economic barrier to a potential shift towards a plant-based diet for consumers adherent to those patterns. Strong adherence to the *Traditional* pattern resulted in a low energy-adjusted carbon footprint but high carbon footprint per euro. This suggests a preference for cheap nutritional energy rather than environment-conscious purchase behaviour. Whether a shift towards a plant-based pattern would be affordable for those with more traditional and cheaper purchase patterns requires more research.

Reducing health risks caused by an unhealthy diet (CHD, type 2 diabetes and cancer (WHO 2013)) and reducing the carbon footprint of food consumption require changes in food consumption patterns^(1)^ which in turn might require changes in food prices^(2,3)^.

Several studies based on theoretical models suggest that changing dietary habits could reduce the carbon footprint of a diet by up to 50–80 %^(4,5)^. Comparisons between diets such as omnivorous, vegetarian and vegan diets only partially reflect the current reality in Western societies, where the proportions of vegetarians and vegans are still low^(6–8)^. Furthermore, we do not know exactly what alternative diets are taking shape and what they contain. To support the climate change mitigation goals and to monitor the effects of any dietary change that is already under way, it is essential to know the carbon footprint of the current food consumption patterns beyond the rarely followed dietary patterns such as vegetarian or vegan, or national averages. In a few studies assessing real-life food consumption patterns, the differences between the carbon footprints of common self-selected dietary patterns have varied from negligible to major^(9,10)^.

To make healthy and environmentally sustainable food available to all, reasonable pricing is important: it can make sustainable food consumption possible for households with low incomes. Several studies suggest that healthy food is more expensive than unhealthy food^(11–14)^. On the other hand, the prices of legumes and grains, considered both healthy and climate-friendly, can be substantially less expensive per kJ than meat^(15)^. Our previous study showed that plant-based protein sources were bought, on average, for a cheaper price than meat^(16)^. In addition, in a US study, vegetarians spent less money on food purchases than meat eaters^(17)^. To make healthy and sustainable foods more attractive to consumers, raising the prices of unhealthy and environmentally unsustainable foods such as red and processed meat could also be a solution^(2,18)^.

Food retailers’ customer loyalty card data provide a unique tool for gaining insights into dietary patterns. We have previously shown that food purchase data are a valid instrument for ranking consumers according to their self-reported food^(19)^ and beer^(20)^ consumption. In this study, the detailed data on purchased product groups over a 1-year period enabled an objective assessment of food expenditure and the allocation of carbon footprints for large sets of product groups on a household level. This automatically accumulating data enabled relatively easy access to unusually large food purchase datasets. Thus, the aim of this study was to identify food purchase patterns by using customer loyalty card data and to study their contribution to the carbon footprints of and expenditure on total food purchases.

## Methods

### Recruitment

**Fig. 1:**
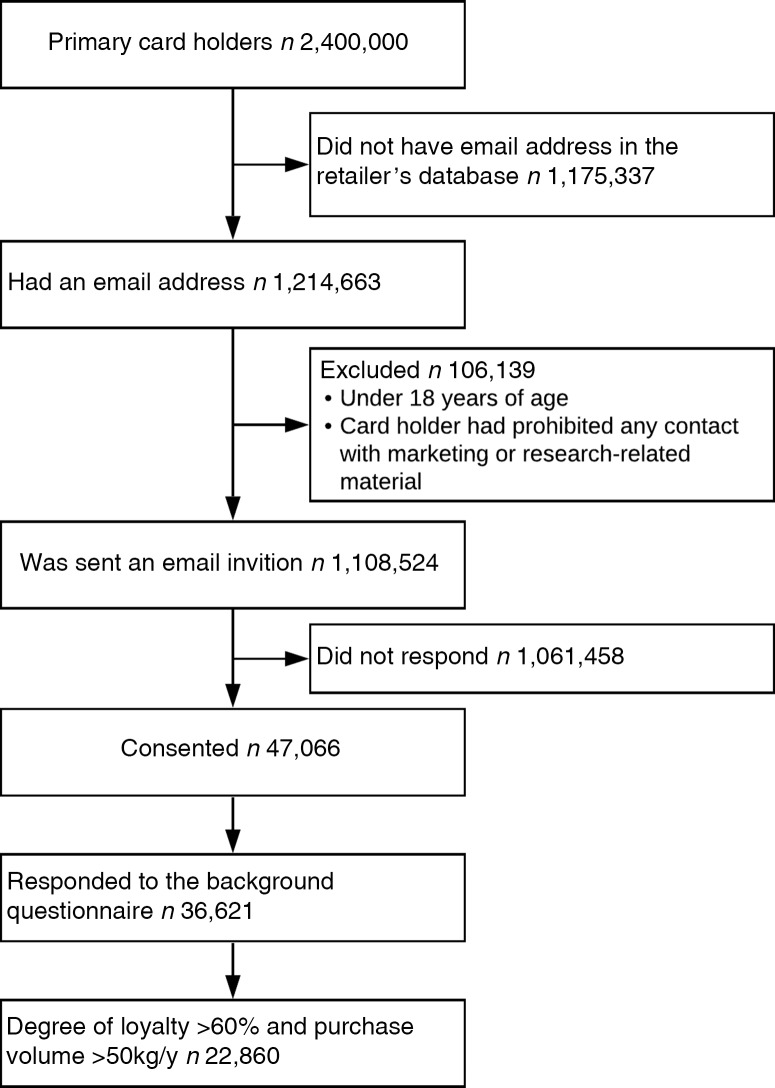
Participant flow

This study utilises large-scale loyalty card data from the largest grocery chain in Finland (S Group)^(21)^. The S Group sells groceries through five retail chains, which are convenience stores, supermarkets, hypermarkets, and one upper-market concept with an extended focus on high-quality and special products. The selection of food items varies between chains, from only a few thousand to over 20 000 items. The retail chains follow an ‘Everyday, low-pricing’ strategy (as opposed to a ‘high–low pricing’ strategy). At the time of the data collection (2018), 2·4 million households in Finland held the S Group’s customer loyalty card, which accounted for 88 % of all Finnish households. Loyalty card holders across Finland received an invitation to the study by email if they had given permission to be approached for research purposes, and if they were aged 18 years or above (Fig. 1). Those who gave their consent for the use of their purchase data for research purposes received an invitation to respond to an additional electronic questionnaire with complementary data on, for example, household structure and income^(22)^.

### Study sample and participants

Initial food purchase data were obtained from *n* 47 066 participants^(22)^. This study comprises data from the year 2018. In the questionnaire, the participants were asked to assess their degree of loyalty to the retailer (i.e. the proportion of purchases from the retailer’s stores of the total food purchases of the household). Only those who had a self-reported degree of loyalty of at least 61 % (i.e. participants who made a large proportion of their food purchases from the food retailer) and who made at least 50 kg of purchases during 2018 were included in the analysis. Our previous analyses showed that purchases associated more strongly with dietary intake among the most loyal (degree of loyalty >60 %) customers^(19,20)^ and therefore build a more complete picture of relative food purchases^(22)^. In this study, we used purchase data aggregated to annual consumption in both volume (kg) and expenditure (€).

### Background data

The retailer’s database provided data on the sex and age of the participants. In the additional questionnaire, the participants reported their number of household members and how many of these were aged 0–6, 7–17, 18–24, 25–64 and 65 years or older. We combined the data on these two questions into a family structure variable that consisted of five categories: single-adult households, one adult and a child/children, two adults, two adults and a child/children, or other (households with three or more adults and households with an unknown family structure). The participants reported their loyalty level to the retailer by choosing from the options of <20 %, 21–40 %, 41–60 %, 61–80 % and >80 %, but as explained in the previous section, only participants in the upper two categories (61–80 %, >80 %) were included in this study.

The participants selected the monthly income of their household from five predefined categories ranging from household income less than 1500 €/month to 9000 €/month or more. Dividing the income (here, the mean of each income category) by the square root of the household size produced the monthly household income (OECD square root scale). This income is thus presented in five categories (less than 1000 €/month, 1000–1999 €/month, 2000–2999 €/month, 3000–3999 €/month and 4000 €/month or more).

### Carbon footprint assessment

**Fig. 2:**
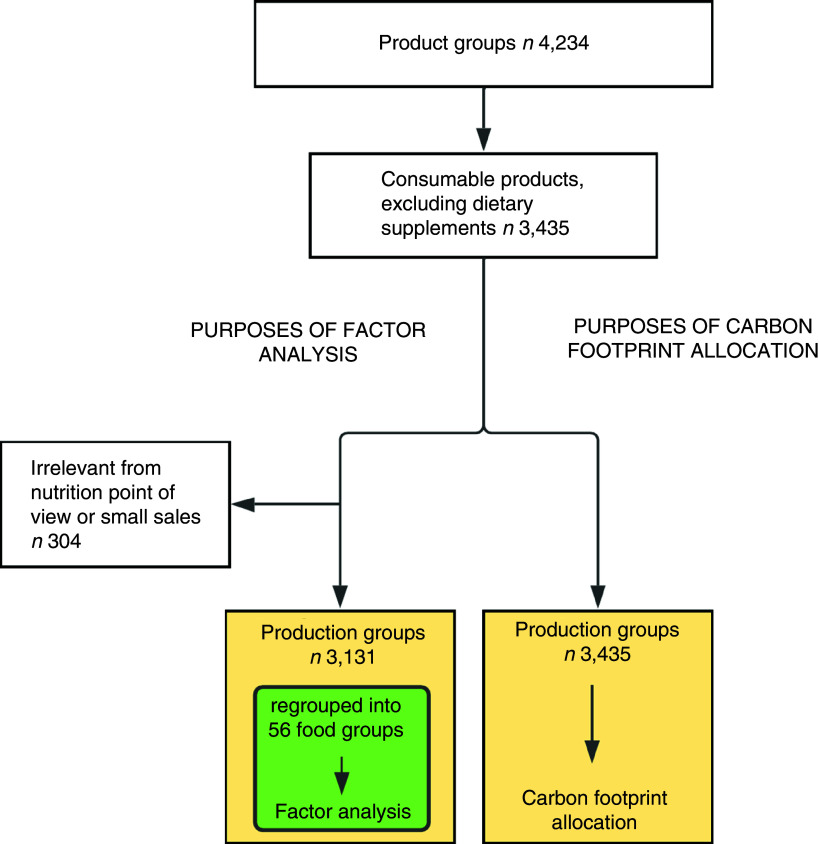
Food item flow

The food retailer originally had 4234 different product groups for their products (Fig. 2). A total of 3435 product groups were assigned a carbon footprint (kg CO_2_-equivalent), using 1 kg of food purchased in retail as the functional unit. As carbon footprint values are not available for all the product groups, indicator products were chosen to represent the 3435 product groups, meaning that one indicator product represented several product groups. Based on the available and suitable LCA studies, we used about 100 different indicator products. As an example of the indicator product approach, all fruits were assigned the same carbon footprint, which was estimated on the basis of the weighted average of the carbon footprints of the five most sold fruits – more than 80 % of the fruits sold. Thus, the carbon footprint of an indicator product stems from carbon footprints of several products. The method for calculating the carbon footprints of the indicator products is described in detail elsewhere (Hartikainen, Heusala, Harrison, Katajajuuri and Silvenius, unpublished results).

The main life cycle phases of the indicator products until retail were included in the system boundaries of the carbon footprint assessment, which comprised the production of inputs to agriculture, agricultural primary production, food processing, packaging, storage (before retail) and transportation. Food waste, land use changes and changes in soil carbon stocks were excluded due to a lack of data.

Data for producing the carbon footprints of the indicator products consisted of a database for food products sold in Finland, compiled by the Natural Resources Institute Finland. The database contains 170 scientific studies of the carbon footprint assessment of food products, and Natural Resources Institute Finland’s LCA database is supplemented with expert estimates. For most of the indicator products, the carbon footprints were medians of the carbon footprints available in data sources. For ready-to-eat meals and beef, additional modifications were made. We determined the meal’s category on the basis of the recipes of the retailer’s most sold meals, using the available carbon footprints for the ingredients. The ‘beef’ category was determined by calculating the carbon footprints of, on the one hand, combined milk and beef production, and on the other hand, suckler beef production from the literature (medians of results from chosen studies), based on how much of the beef was sourced from the combined and suckler beef production systems^(23)^. The data for storing, according to the three options of dry, cold and frozen, were from the EcoInvent database^(24)^, and the data for packaging were from Plastics Europe^(25)^, the European Aluminium Association^(26)^, the World Steel Association^(27)^ and the FEFCO^(28)^. The distance between the producer country and the logistics centres needed for the assessment of emissions from transportation were calculated based on six main production areas: (1) Finland; (2) other Nordic countries and Estonia; (3) the rest of Europe; (4) America; (5) Africa and the Middle East; and (6) Asia. The emission factors for transportation were taken from the Lipasto database^(29)^, except for the trans-oceanic container ship, which was from the Ecoinvent database^(24)^.

After the carbon footprints of the indicator products had been determined for the retailer’s product groups, the purchase volume of each loyalty card holder for each product group (kg) was multiplied by the corresponding carbon footprint (kg CO_2_-eq) to obtain customers’ product group-specific and total-purchase carbon footprints. The sum of the carbon footprints of all the product groups represents the carbon footprint of the total food purchases per person.

### Nutritional energy content of the purchases

Throughout the text, energy refers to the nutritional energy content of the food purchases (not, e.g., energy utilised in food production). The energy content of 1 kg of each product group (e.g. cucumber, skimmed milk and vegetarian lasagna) was derived from the nutrition calculation software on www.fineli.fi. This webpage utilises the food composition database Fineli which is maintained by the Finnish Institute for Health and Welfare. The purchase volume (kg) of each product group was multiplied by the energy content per 1 kg of the group to obtain the absolute energy content of the purchase. The energy contents of all the purchased product groups were summed to obtain the annual energy content of the total purchases.

### Grouping of food purchase data for factor analysis

For factor analysis, a major regrouping was conducted, based on the purpose of use (combined fresh vegetables such as cucumber, tomato etc., as fresh vegetables; soya milk, soya yoghurt, oat milk, oat yoghurt etc., as different plant-based dairy alternatives, etc.). The aggregation of food groups was restricted to a level that enabled differentiation on the basis of nutritional content and carbon footprint. This was driven by the differences in nutrient content that are relevant for public health in Finland and that are reflected in the food-based dietary guidelines of the Nordic Nutrition Recommendations (e.g. separating high-fibre from low-fibre breads and high-fat from low-fat dairy), the degree of processing (e.g. separating fresh potato from frozen potato) and the carbon footprints of the product groups (e.g. separating meat types such as beef, pork and poultry) (see online Supplemental Table 1). Examples of the aggregated food groups and product groups included were as follows: ‘skimmed milk and sour milk’: regular, low-lactose, and lactose-free skimmed milk and skimmed sour milk, and ‘sugar-sweetened beverages’: soft drinks, energy drinks, juices, ice teas and seasonal drinks. Product groups that were not relevant to overall diet quality (e.g. tea, bottled water, chewing gum and spices) or product groups with very low purchases (e.g. game, reindeer and horse meat) were excluded. The final number of food groups to be used in factor analysis was 56.

### Statistical methods

Participants’ characteristics are presented as means and standard deviations, or frequencies and percentages.

To estimate the total household food purchases, we multiplied the volume of total purchases (kg) by the inverse of the self-reported degree of loyalty, for which we used the midpoints of the category intervals. Deviation from normal distribution, detected by visual inspection of their empirical distributions, led to the logarithmic transformation of the food group variables, the energy content of the total food purchases, the carbon footprint (CO_2_-eq. values) of and expenditure (€) on total food purchases and the carbon footprint to expenditure ratio. Before the subsequent factor analysis on food purchases measured in kilograms, we performed a 98 % winsorisation of the food group variables to diminish the effect of outliers, that is, outliers below the 1st percentile and above the 99th percentile were truncated into the 1st and 99th percentile, respectively.

Bartlett’s test of sphericity (*χ*^2^(1540) = 567 540, *P* < 0·001) suggested the appropriateness of the factor analysis and the Kaiser–Meyer–Olkin test (KMO = 0·96) indicated good sampling adequacy. Food purchase patterns were derived from principal component analysis, based on the correlation matrix of food groups^(30)^. The number of principal components was decided by simultaneously examining the scree plot (see online Supplemental Fig. 1), the Kaiser criterion (eigenvalue >1), the percentage of explained variation (our aim was >50 %) and the interpretability of the factors. We chose eight components and used an orthogonal varimax rotation to produce the final factors, from which we then identified and named the food purchase patterns. All the participants were assigned standardised factor scores to represent food purchase patterns, that is, weighted combinations of the purchased food groups. The pattern scores showed how strongly empirically derived purchase patterns (and the food groups defining it) were reflected in a participant’s shopping basket; the higher the score, the stronger the adherence to the purchase pattern. As customary in nutrition research, the patterns were named based on a feature that was common for the food groups that had high loadings for the factor and that separated the factor from the other factors.

The associations between the food purchase patterns and carbon footprint or expenditure were analysed using linear regression analysis with purchase pattern scores as explanatory variables and log-transformed carbon footprints or log-transformed expenditure as the response variable. The model had one purchase pattern at a time and the log-transformed energy content of the total purchases as explanatory variables, meaning that each pattern was analysed separately. Thus, as the analyses were adjusted for the (log-) energy content, the regression coefficients can be interpreted as the difference between the log-carbon footprint or log-expenditure of two individuals with the same energy content of the total purchases but a unit’s (sd) difference in their purchase pattern. To illustrate the magnitude of the effects of the patterns in a more perceivable manner, we calculated the estimated carbon footprint and expenditure in the lowest and highest thirds and in the lowest and highest 10 % of the pattern scores of each pattern using the regression equation:

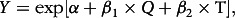

where Y is either carbon footprint or expenditure, α is the intercept term, *β*_1_ equals the regression coefficient of the pattern score, Q equals the mean of the pattern score in a given quantile (lowest third, highest third, lowest 10 % or highest 10 %) and *β*_2_ equals the regression coefficient of the log-transformed energy content at its mean value (T).

The associations between the patterns and the ratio of carbon footprint (kg CO_2_-eq.) to expenditure (€) were analysed using simple regression analysis, of which the log-transformed carbon footprint:expenditure ratio was the outcome and one pattern at a time an explanatory variable. The patterns that could not be considered overall dietary patterns, namely *Skimmed milk and margarine* and *Alcohol*, were excluded from further analyses.

To gain further insights into the association between purchase patterns and carbon footprint and expenditure, we calculated (1) the individual food groups’ sum of the annual carbon footprint (kg CO_2_-eq.), (2) the percentage of each food group’s carbon footprint of the annual total, (3) the sum of annual expenditure (€) on the individual food groups, (4) the percentage of expenditure on each food group of the total annual expenditure, and (5) the ratio of the annual carbon footprint and the annual expenditure (see online Supplemental Table 2).

### Sensitivity analyses

To investigate the sensitivity of different decisions to the result of the factor analysis, we conducted factor analysis with several different choices: (1) included only participants with an ≥80 % degree of loyalty; (2) no winsorisation of the food variables before factor analysis and (3) 5 % winsorisation of the food variables before factor analysis. The results of these factor analyses were similar to the one presented; the same patterns with similar explained variations were identified. Therefore, these results are not shown.

## Results

The majority of the participants were women (66 %) (Table 1). A two-adult household was the most common family structure (34 %), followed by single-adult households (25 %), and two adults with a child/children (23 %). The majority of the households fell into the scaled monthly income range of 2000–2999 € or 3000–3999 € (29 % and 23 %, respectively), and the majority (61 %) bought 81–100 % of all of their food purchases from the retailer.

**Table 1:** Background characteristics of participants (*n* 22 860)

	*n*	%
Sex		
Men	7745	33·9
Women	15 115	66·1
Age		
	Mean	sd
	47·9	15·2
	*n*	%
Family structure		
Single-adult households	5717	25·0
One adult and a child/children	1040	4·5
Two adults	7875	34·4
Two adults and a child/children	5272	23·1
Other	1707	7·5
Missing	1249	5·5
Scaled household income (€/month)*		
Less than 1000	1967	8·6
1000–1999	3398	14·9
2000–2999	6633	29·0
3000–3999	5147	22·5
4000 or more	4237	18·5
Missing	1478	6·5
Percentage of purchases from retailer		
61–80 %	8978	39·3
81–100 %	13 882	60·7
	Median	IQR
CO_2_ of total food purchases from S Group (kg CO_2_-eq/year)		
	2750	1676, 4339
Total purchase volume (kg/year)		
	733	463, 1129
Energy content of the total purchases (MJ/year)		
	4244	2721, 6506
Expenditure on food (€/year)		
	3141	2015, 4691

* Income (here the mean of each income category) divided by the square root of the household size produced (OECD square root scale).

### Purchase patterns

**Fig. 3:**
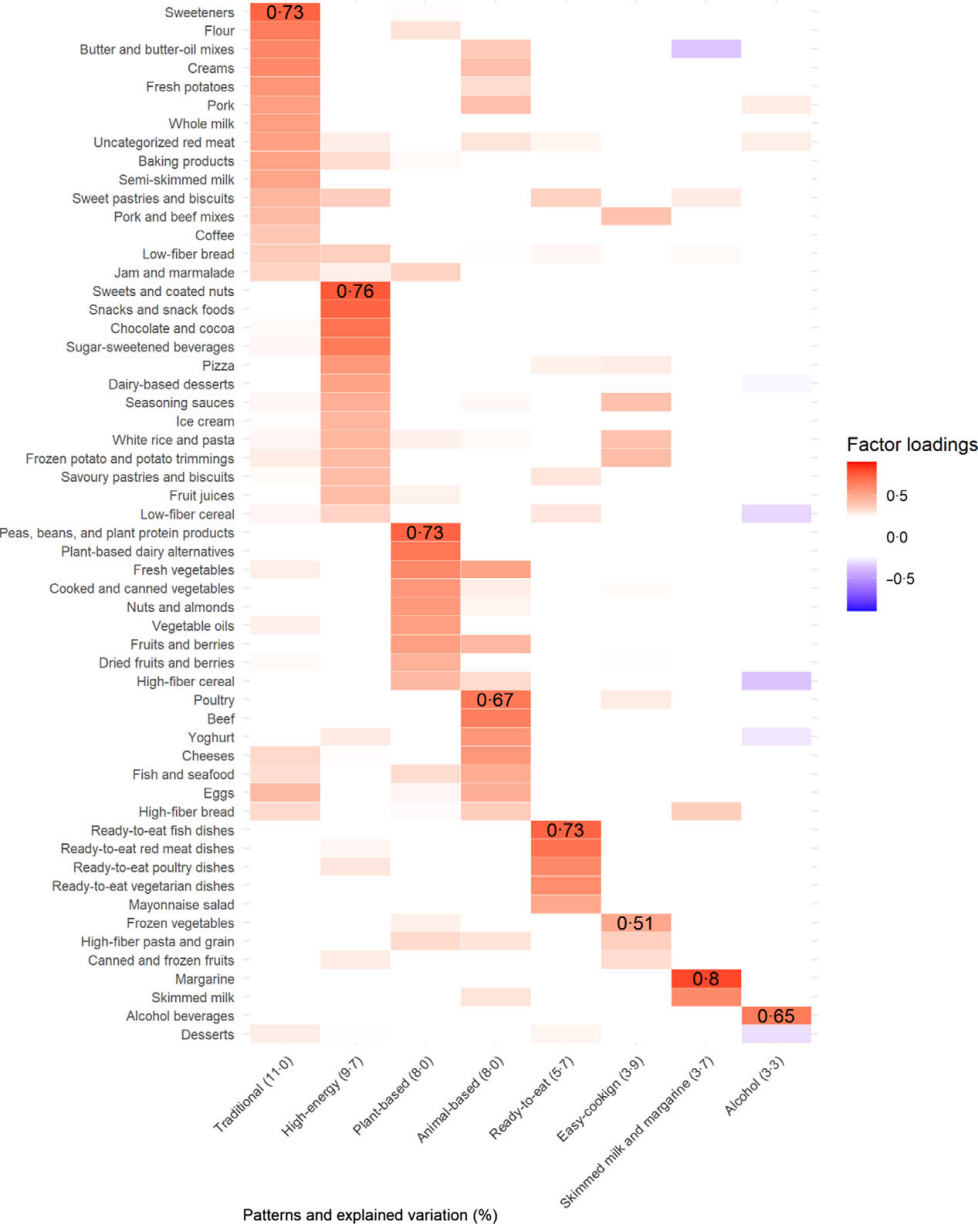
Illustration of rotated principal components’ loading matrix of food purchase patterns. The values in the tiles represent the largest factor loading within each pattern. The percentages of explained variances for the factors are in parenthesis after the pattern names under the x-axis

Eight factors were derived, which explained altogether 55 % of the variation of the fifty-six food groups (Fig. 3). In descending order of explained variation, the patterns were named *Traditional* (11·0 % of variation)*, High-energy* (9·7 %)*, Plant-based* (8·0 %)*, Animal-based* (8·0 %)*, Ready-to-eat* (5·7 %)*, Easy-cooking* (3·9 %)*, Skimmed milk and margarine* (3·7 %) and *Alcohol* (3·3 %). Figure 3 shows the food group loadings in each of the patterns.

### Carbon footprint of the purchases and association with purchase patterns

An investigation of the food groups behind the patterns showed that 12 % of the total carbon footprint of the purchases originated from beef and processed beef and 9 % from cheese (see online Supplemental Table 2). Both food groups were strongly loaded in the *Animal-based* pattern (factor loading for beef and processed beef 0·65). The third largest food group that contributed to the total carbon footprint was fresh vegetables (6 %), which loaded strongly in the *Plant-based* (factor loading 0·61) and *Animal-based* (0·51) patterns. In contrast, peas, beans and lentils, which loaded strongly (0·73) in the *Plant-based* pattern, made only a small contribution to the total carbon footprint (0·4 %). All the meat food groups together contributed to 29 % of the total carbon footprint, all the dairy food groups to 28 %, and all the vegetables, fruits and berries to 12 % of the total carbon footprint.

When adjusted for the energy content of the annual purchases, the difference in the carbon footprint (log-kg CO_2_-eq.) of 1 sd difference in pattern scores was the largest for the *Animal-based* pattern (*β* 0·134, 95 % CI (0·132, 0·137)), followed by *Easy-cooking* (*β* 0·039, 95 % CI (0·036, 0·042)) and *Ready-to-eat* (*β* 0·016, 95 % CI (0·013, 0·019)) (Table 2). For the *High-energy* (*β* −0·032, 95 % CI (−0·035, −0·029)), *Traditional* (*β* −0·036, 95 % CI (−0·039, −0·032)) and *Plant-based* patterns (*β* −0·047, 95 % CI (−0·050, −0·044)), the relationship was inverse; a 1 sd higher *Plant-based* score was associated with a significant decrease in the carbon footprint of the total purchases. In other words, the *Animal-based, Easy-cooking and Ready-to-eat* patterns were positively associated, and the *Plant-based, Traditional* and *High-energy* patterns were inversely associated with the carbon footprint of the total purchases.

**Table 2:** Regression coefficients (*β*) and 95 % CI for association between food purchase patterns and log-transformed annual carbon footprint with energy from the purchases (MJ) at its annual mean level, and predicted carbon footprint (kg CO_2_-eq/year) in the lowest (T1) and highest thirds (T3), and lowest (D1) and highest deciles (D10) of each purchase pattern

Pattern	*β*	95 % CI		D1	T1	T3	D10
		Lower	Upper				
Animal-based	0·134	0·132	0·137	2119	2225	2970	3218
Easy-cooking	0·039	0·036	0·042	2437	2471	2691	2761
Ready-to-eat	0·016	0·013	0·019	2529	2539	2625	2663
High energy	−0·032	−0·035	−0·029	2701	2671	2488	2437
Traditional	−0·036	−0·039	−0·032	2710	2680	2477	2415
Plant-based	−0·047	−0·05	−0·044	2743	2706	2446	2349

The comparison of the highest 10 % of *Animal-based v*. *Plant-based* revealed a + 869 kg CO_2_-eq annual difference, which means a 27 % lower annual food purchase carbon footprint among those strongly adhering to the *Plant-based* pattern than among those strongly adhering to the *Animal-based* pattern. Those with the highest 10 % of *Easy-cooking* and *Ready-to-eat* scores had a 14 % and 17 % smaller carbon footprint, respectively, than those in the highest 10 % of the *Animal-based* scores, but *Easy-cooking* and *Ready-to-eat* had a 17 % and 13 % larger carbon footprint, respectively, than those with the highest 10 % of the *Plant-based* scores. The carbon footprint of those in the highest 10 % of *High energy* and in the highest 10 % of *Traditional* was close to that of the *Plant-based* pattern, only 4 % and 3 % higher, respectively.

### Food expenditure and association with purchase patterns

An investigation of the food groups behind the patterns showed that of the food groups, expenditure was highest on cheese (7 %), fresh vegetables (7 %), fruits and berries (6 %), alcohol beverages (6 %), and yoghurt (5 %) (see online Supplemental Table 2). Cheese and yoghurt were strongly loaded in the *Animal-based* pattern, whereas fresh vegetables, and fruits and berries were strongly loaded in the *Plant-based* and *Animal-based* patterns. Peas, beans and lentils made up only 0·65 % of the total food expenditure.

When adjusted for the annual energy content of the purchases, all the purchase patterns were associated with the total expenditure on food (log- €) (Table 3). The regression indicated a positive correlation for all patterns except those of *Traditional* (*β* −0·115, 95 % CI (−0·120, −0·111)) or *Easy-cooking* (*β* −0·029, 95 % CI (−0·033, −0·025)), for which the correlations were inverse. The change in the expenditure on food purchases by a 1 sd increase in the food purchase pattern score (adjusted for total energy content of the purchases) was the largest and inverse in the *Traditional* pattern and the second, third, and fourth largest in the *Animal-based* (*β* 0·064, 95 % CI (0·059, 0·068))*, Ready-to-eat* (*β* 0·063, 95 % CI (0·059, 0·066)) *and Plant-based* (*β* 0·055, 95 % CI (0·051, 0·059)) patterns, but in the opposite direction to that of *Traditional*. Comparison of the highest 10 % of the *Traditional **v***. *Animal-based* patterns revealed a + 886 € annual difference between the patterns, which means a 27 % lower annual expenditure among those in the highest 10 % of the *Traditional* pattern ***v***. those in the highest 10 % of the *Animal-based* pattern. The difference between those in the highest 10 % of *Traditional* and *Plant-based* was similar.

**Table 3:** Regression coefficients and 95 % CI for association between food purchase patterns and log-transformed annual expenditure on food (€) with energy from the purchases (MJ) at its annual mean level, and predicted expenditure (€) in the lowest (T1) and highest thirds (T3), and lowest (D1) and highest deciles (D10) of each purchase pattern

Pattern	*β*	95 % CI		D1	T1	T3	D10
		Lower	Upper				
Animal-based	0·064	0·059	0·068	2685	2748	3151	3273
Ready-to-eat	0·063	0·059	0·066	2730	2770	3164	3353
Plant-based	0·055	0·051	0·059	2742	2786	3134	3285
High energy	0·029	0·024	0·033	2828	2855	3042	3098
Easy-cooking	−0·029	−0·033	−0·025	3072	3041	2855	2801
Traditional	−0·115	−0·12	−0·111	3458	3336	2588	2387

### Relationship between carbon footprints and expenditures

When the food groups behind the patterns were examined separately, the largest carbon footprint per euro was for beef and processed beef (2·6), followed by pork and beef mixes (2·1), skimmed milk and sour milk (1·3), butter and butter–oil mixes (1·2), and semi-skimmed milk and sour milk (1·1) (see online Supplemental Table 2). In contrast, peas, beans and lentils had small carbon footprints per euro (0·34). Pork and beef mixes, both milk food groups, and butter–oil mixes loaded strongly in the *Traditional* pattern.

**Fig. 4:**
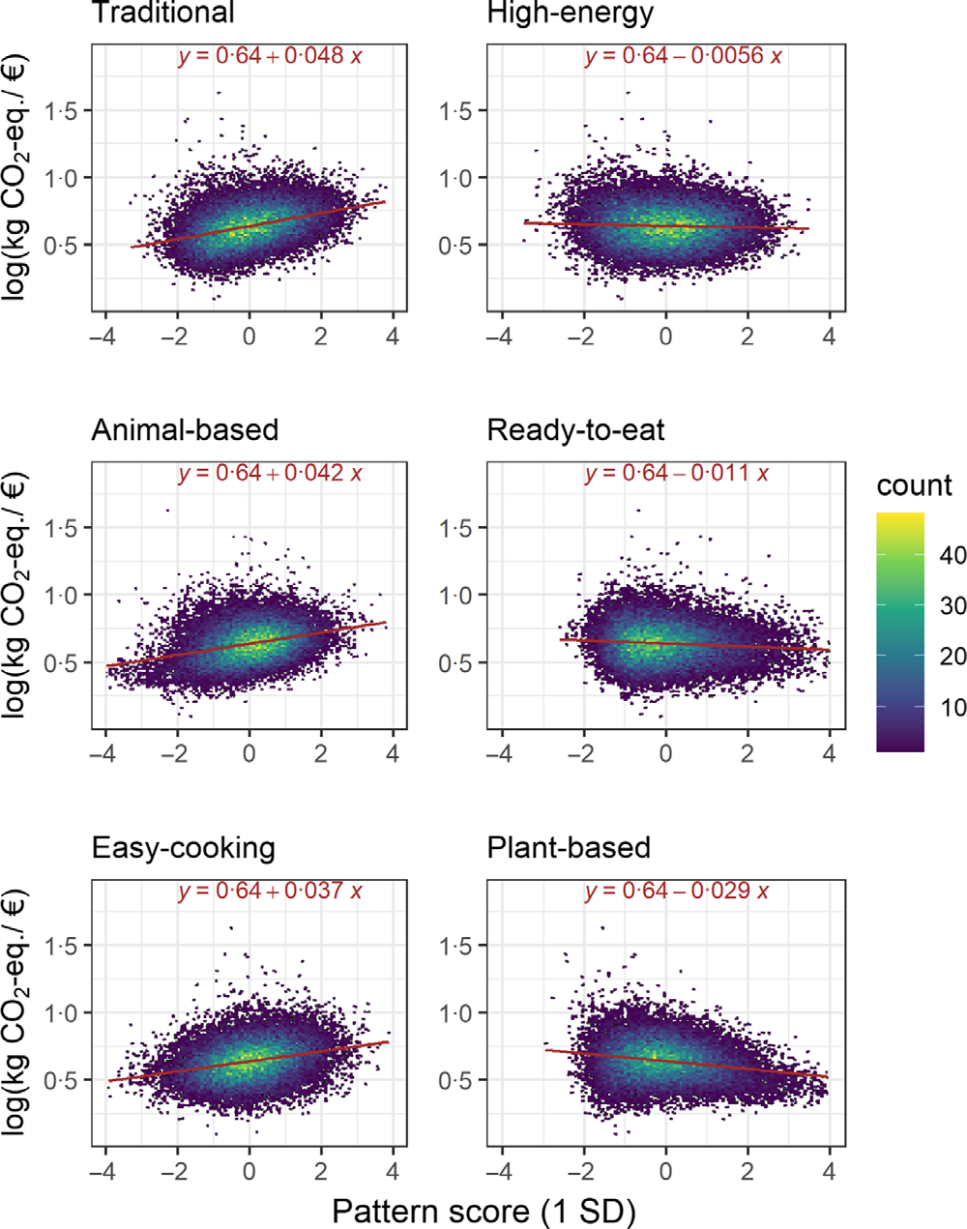
Relationship between the purchase patterns and the log-transformed ratio of carbon footprint (kg CO_2_-eq.) and expenditure (€)

When adjusted for the energy content of the annual purchases, total expenditure (log-€) was positively associated with total carbon footprint (log-kg CO_2_-eq., *β*: 0·36, 95 % CI (0·35, 0·37)). Figure 4 shows the relationships between the patterns and the ratio of carbon footprint and expenditure. Stronger adherence to the *Traditional* (*β*: 0·048, 95 % CI (0·047, 0·050)), *Animal-based* (*β*: 0·042, 95 % CI (0·040, 0·044)) and *Easy-cooking* (*β*: 0·037, 95 % CI (0·036, 0·039)) patterns were associated with a higher carbon footprint per spent euro, whereas stronger adherence to the *High-energy* (*β*: −0·006, 95 % CI (−0·007, −0·004))*, Ready-to-eat* (*β*: −0·011, 95 % CI (−0·013, −0·010)) and *Plant-based* (*β*: −0·029, 95 % CI (−0·030, −0·027)) patterns were associated with a lower carbon footprint per spent euro.

## Discussion

We identified eight food purchase patterns, from which we further analysed the six that explained most of the variation. Of all the patterns, the *Animal-based* explained the carbon footprint of total food purchases the most, that is, the purchases of those strongly adhering to the *Animal-based* had the largest carbon footprint, followed by *Easy-cooking* and *Ready-to-eat* patterns. As expected, the purchases of those who adhered strongly to the *Plant-based* pattern had the smallest carbon footprint. Those who adhered strongly to the *Animal-based, Ready-to-eat* and *Plant-based* patterns spent the most money on food, whereas those who adhered strongly to the *Traditional* pattern spent the least money on food. Stronger adherence to the *Traditional, Animal-based* and *Easy-cooking* patterns was associated with a larger carbon footprint per euro spent. This was because these patterns had high loadings of animal-based food groups, which had high carbon footprint to expenditure ratios.

Prior research has used alternative methods such as bar-code scanning to study purchase patterns^(31–33)^, and only one has used customer loyalty card data^(34)^. Because loyalty card data accumulate without any effort required from the participant and is provided by the retailer instead of the customer/participant, they are possibly more objective than data collected by participants using bar-code scanning. Automated accumulation of data without much effort from researchers or participants enables data from a larger number of participants than data collected by participants using bar-code scanning. A detailed comparison of our study and previous studies of purchase patterns *per se* is not feasible because the countries of these studies have different food cultures (Finland, UK, Germany and USA), the analysed food groups consist of different foods and the analytical methods are different. However, one German^(33)^ and one US study^(31)^ found patterns similar to our *Traditional* pattern, characterised by high loadings for vegetables, fruits, potatoes, high-fat milk, and high-fat meat, and the US study^(31)^ found a pattern characterised by ready-to-eat meals, and a pattern characterised by sweets, snacks and deserts. These patterns resembled our *Ready-to-eat* and *High-energy* patterns, respectively.

A few studies have analysed carbon footprints by using data-driven food consumption patterns, but not actual purchase data^(10,35,36)^. Comparing our study to somewhat similar studies is challenging because of differing food consumption assessment methods (dietary intake *v*. food purchases), differing covariates in the models (e.g. energy adjustment), varying LCA methodologies, the individual method choices of assessing CO_2_-eq values (allocations, system boundaries, etc.), and different food production conditions and practices. All these lead to the studies having different carbon footprints. It is also important to note that unlike dietary intake data, food purchase data also include foods that end up in household food waste (an advantage when studying the environmental impacts of food consumption). The direction of the results of the most similar study to ours^(35)^, however, resembled the direction of ours: a food consumption pattern characterised by a high consumption of meat was associated with a larger carbon footprint than the patterns characterised by less consumption of meat. In the same study, the carbon footprints of the patterns characterised by less meat consumption, such as a plant-based healthy pattern (Lebanese-Mediterranean pattern) and a pattern with high loadings for high-energy/low-nutrient foods, were not fundamentally different to each other. This was similar to our result showing that the carbon footprints of *High-energy* and *Plant-based* patterns differed only a little.

The small size of the carbon footprint related to strong adherence to the *High-energy* pattern, which explained the variation the second most, can be explained by its high loadings for only plant-based foods. These foods, however, were typical ultra-processed foods^(37)^, which are high in sugar, saturated fat and energy, and low in fibre and micronutrients^(38,39)^. The consumption of ultra-processed foods is associated with obesity and other non-communicable diseases^(40–42)^, although it has not been conclusively shown that these adverse health effects are due to ultra-processing *per se*. Thus, despite a small carbon footprint, a *High-energy* pattern is not recommendable as a sustainable alternative to patterns with a large carbon footprint.

The relative increase in the *Ready-to-eat* score was associated with only a moderate increase in the carbon footprint of total food purchases. This was probably because the *Ready-to-eat* pattern is a mixture of animal- and plant-based foods (vegetarian, red meat, poultry and fish), and in Finland, the red meat alternatives of ready-to-eat meals usually contain relatively small quantities of meat. Data on the carbon footprints of ready-to-eat meals are scarce, however, and the results of earlier studies vary. In a Finnish study, ready-to-eat meals had a smaller carbon footprint than home-cooked equivalents, owing to raw material selection in ready-to-eat meals^(43)^. In contrast, in a UK study, ready-to-eat meals had a greater carbon footprint than equivalent home-cooked meals, mainly due to higher waste production during the processing phase^(44)^. Our data on ready-to-eat meals are based on the scarce available LCA data, which is why any interpretation of the carbon footprint of *Ready-to-eat* pattern requires caution. More data on the carbon footprints of ready-to-eat meals are clearly required.

According to nutrition recommendations, the food groups to be consumed the most are fruits, vegetables and high-fibre grains^(45)^. Furthermore, given the climate mitigation goals, consumption and purchases of plant-based products should be much more common, particularly if it leads to a reduction in meat consumption. Previous studies suggest that a plant-based sustainable diet is not affordable for everyone, especially in low- and middle-income countries^(46,47)^. Compared to other food groups, fruits and vegetables were expensive, whereas starchy staple foods (e.g. wheat flour, potatoes and rice) were the least expensive in all regions of the world^(46)^. Previous studies, however, have not extensively investigated the expenditure on food of those adhering to plant-based consumption patterns in developed countries.

In terms of reducing the carbon footprints of the households in our study sample, a shift from *Animal-based and Easy-cooking* patterns towards *Plant-based* pattern would be beneficial. Those adhering strongly to the *Animal-based* pattern (highest 10 % of the total pattern score) spent similar amount of money on food as those adhering strongly to the *Plant-based* pattern, which suggests a lack of economic barrier for a necessary shift towards a plant-based pattern. Those adhering strongly to *Easy-cooking* spent 484 €/year less on food than those adhering strongly to the *Plant-based* pattern. The carbon footprint of those adhering strongly to the *Traditional* pattern was not much larger than that of those strongly adhering to the *Plant-based* pattern, and more plant-based food choices would improve the nutritive value of their purchases. The difference between the expenditures of those adhering strongly to the *Traditional* and those adhering strongly to the *Plant-based* pattern was great – 898 €/year. However, it is worth noting that purchase data are based on actual expenditures. They do not represent the cheapest or most expensive selections. Therefore, cheaper, healthy plant-based food baskets are probably available. What they would contain and on what terms they would appeal to consumers requires more research. Thus, our results suggest that a shift from the *Animal-based* to the *Plant-based* pattern should not be considered as an economic issue. An economic barrier to shifting from the *Traditional* and *Easy-cooking* to a healthy plant-based pattern would be an important research topic.

Those strongly adhering to *High-energy* foods had a relatively high expenditure on food. Because of the unhealthy characteristics of ultra-processed foods, the health authorities usually consider ultra-processed foods, which are typically considered cheap, a threat to the health of the lowest income households in particular^(30)^. Our results suggest that the expenditure on food of at least those who adhered strongly to the *High-energy* pattern was near the average among the loyalty card holders. High palatability, affordability, convenience (often sold as ready-to-consume) and effective marketing may also increase the purchases of *High-energy* foods among those with higher expenditure. Even more so than *High-energy,* the *Ready-to-eat* pattern was not associated with low expenditure on food, which suggests that aiming for low expenditure is not the main motive of purchasing ready-to-eat meals. Our results thus suggest that a shift from *Ready-to-eat* pattern to a *Plant-based* pattern might not be an economic issue. This is supported by a previous study, which showed that convenience was an important food motive among Finnish consumers, especially among younger individuals and households with adults and children^(48)^. Based on these results, one way to acknowledge the convenience motive but to improve healthiness and reduce the carbon footprint of food consumption could be to increase the availability of healthy plant-based ready-to-eat meals with small carbon footprints.

In our analyses of the carbon footprint per euro in relation to the patterns, the most important results were those of the *Traditional* and *Ready-to-eat* patterns. Although those adhering strongly to the *Traditional* pattern had a lower carbon footprint than the other patterns, their carbon footprint per euro was high. This may indicate primarily cheap-energy-driven rather than environment-conscious purchase behaviour, resulting in an unintentional outcome of a smaller carbon footprint than among the others for the same total energy content. For those strongly adhering to the *Ready-to-eat*, the carbon footprint per euro was somewhat low, which is logical because they had an average carbon footprint but high expenditure on food. The carbon footprints per euro in relation to the other patterns were mostly in line with the energy-adjusted carbon footprints related to the patterns; those with a high carbon footprint, *Animal-based* and *Easy-cooking*, also had a high carbon footprint per euro and those with a low carbon footprint, *Plant-based* and *High-energy*, had a low carbon footprint per euro.

This study has several strengths. The potential of customer loyalty card data for investigating food purchase behaviour patterns has remained largely unexplored. Unlike self-reported food consumption data, customer loyalty card data do not suffer from recall bias or under- and over-reporting. Extensive purchase data are obtainable without substantially burdening participants or researchers. As the data covered an extensive time period, they were more accurate regarding habitual consumption and the inclusion of the seasonal variation of food consumption. We have also previously shown that food purchase data are a valid instrument for ranking consumers according to their self-reported food^(19)^ and beer^(20)^ consumption. Our data included detailed information on thousands of product groups, which enabled regrouping based on the principles most appropriate for the purpose. No studies have analysed carbon footprints or the expenditure of data-driven food purchase patterns. Conclusively, our study is among the first to display how customer loyalty card data can be used to identify and assess purchase patterns and the associated carbon footprints and expenditure.

Some uncertainties regarding the data are worth discussing. Even though most of the purchases were bought from the retailer in question, some foods may have been bought from different retailers, especially by those who reported buying only 61–80 % of their groceries from the retailer. However, our sensitivity analysis showed that the purchase patterns were similar when only those who bought ≥81 % of their food from the retailer were included in the factor analysis. This is in line with our previous finding that the proportions of food groups purchased were very similar among customers with high loyalty^(22)^, and that their purchases reflect the loyalty card holder’s dietary intake^(19)^. To estimate the similarity of the purchase data with the purchases of the general Finnish population, the average annual expenditure on groceries and non-alcoholic beverages in Finland was 2916 €, and on alcohol and cigarettes 578 € in 2016^(49)^. These figures are not completely comparable to our expenditure data (median 3141 €/year) because the food expenditure in our data was only that of the primary card holder, because only alcoholic beverages with ≤5·5 % of alcohol are available in grocery stores in Finland, because our study data did not include cigarettes, and because of inflation (0·72 % from 2016 to 2018). These figures are, however, of somewhat similar magnitude.

Finally, the study also had some limitations. The sample was selected as those who bought most of their grocery shopping from the retailer of the present study and excluded those who preferred other retailers. The customers of different retailers may have different background or purchase profiles. As we have shown earlier, the purchase sample differed slightly from that of the general Finnish population; there were more women, individuals with higher education, and employed individuals, and less individuals aged under 30 years and over 70 years, as well as retired individuals^(22)^. It is therefore possible that the purchase patterns specific only to men, to those with lower education, unemployed, and to those aged under 30 or over 70, may not have been identified by the factor analysis. In the indicator product approach, some categories contained versatile food items, which increases the uncertainty related to the carbon footprint estimates. For instance, carbon footprints for ready-to-eat meals should be considered as rough estimates. However, the indicator product approach enabled reasonably sophisticated estimates, and the results of the study can be considered robust estimates of the relative differences concerning carbon footprint of the purchase patterns. The carbon footprints of product groups did not include the customer phase, which could have a significant impact on the carbon footprints of the purchases due to, for example, transportation from stores to homes or cooking methods. It should be noted that we only covered carbon footprints, and hence other highly relevant environmental impacts of food purchase patterns (e.g. biodiversity, water footprint, eutrophication and acidification) were not assessed, although all environmental impacts should be considered together when planning actions, due to their potential trade-offs.

## Conclusions

The carbon footprint was the greatest in those with strong adherence to the *Animal-based* and the lowest in those with strong adherence to the *Plant-based* pattern. The finding that strong adherence to the *Traditional* pattern resulted in a low energy-adjusted carbon footprint but high carbon footprint per euro suggests primarily cheap-energy-driven rather than environment-conscious purchase behaviour. The *High-energy* and *Ready-to-eat* patterns, which were both associated with moderate carbon footprints, associated with high expenditure, suggesting motives other than aiming for minimising expenditure. Because those adhering strongly to the *Animal-based* and the *Plant-based* patterns spent nearly equivalent amounts of money on food, a shift towards a recommended plant-based purchase pattern would probably not be an economic issue for those strongly adhering to the *Animal-based* pattern. The characteristics of affordable, healthy, plant-based purchase patterns that would be appealing to those who spend less money on food require further research.

## Supplementary material

For supplementary material accompanying this paper visit https://doi.org/10.1017/S1368980022001707. click here to view supplementary material
