# Food purchase behaviour in a Finnish population: patterns, carbon footprints and expenditures – ADDENDUM

**DOI:** 10.1017/S1368980023000563

**Published:** 2023-05

**Authors:** Jelena Meinilä, Hanna Hartikainen, Hanna L Tuomisto, Liisa Uusitalo, Henna Vepsäläinen, Merja Saarinen, Satu Kinnunen, Elviira Lehto, Hannu Saarijärvi, Juha-Matti Katajajuuri, Maijaliisa Erkkola, Jaakko Nevalainen, Mikael Fogelholm

The authors regret the incorrect expression of “total expenditure” in the above article.

“Expenditure on total food purchases” was used in the article, when in fact the expenditure included, on top of food products, groceries such as shampoo, plastic bags, and tobacco products. The proportion of the non-food groceries from the total expenditure was on average 17 %.

To examine whether inclusion of the non-food items would affect the interpretations of the study, the authors have repeated the analyses leaving out the non-food items. They found that the interpretations of the results did not change substantially, i.e. the regression coefficients for the association between food purchase patterns and log-transformed annual total expenditure (adjusted for annual energy content of the purchases) changed only little when non-food items were omitted, although the predicted expenditure in the thirds and deciles of the pattern scores were lower across the purchase patterns. The interpretations from the analyses concerning the ratio of carbon footprint and expenditure did not change substantially.
