# Supplementary material for: Food purchase behaviour in a Finnish population: patterns, carbon footprints and expenditures
Source: Public Health Nutr. 2022 Aug 18;25(11):3265–77. doi: 10.1017/S1368980022001707 (PMC9991547; doi:10.1017/S1368980022001707)
Supplement: Supplementary file 1 [file S1368980022001707sup001.docx]

| **Supplementary table 1.** The 56 food groups utilized in factor analysis. | |
| --- | --- |
| Butter and butter-oil mixes | High-fiber pasta and grain |
| Canned and frozen fruits | Eggs |
| Cheeses | Fish and seafood |
| Coffee | Peas, beans, and plant protein products |
| Cooked and canned vegetables | Plant-based dairy alternatives |
| Creams | Flour |
| Desserts | Chocolate and cocoa |
| Dried fruits and berries | Semi-skimmed milk and sour milk |
| Ready-to-eat fish dishes | Skimmed milk and sour milk |
| Frozen vegetables | Whote rice and pasta |
| Fruit juices | Frozen potato and potato trimmings |
| High-fiber bread | Sweets and coated nuts |
| High-fiber cereal | Nuts and almonds |
| Ice cream | Beef and processed beef |
| Jam and marmalade | Pork and processed pork |
| Low-fiber bread | Poultry and processed poultry |
| Low-fiber cereal | Uncategorized red and processed meat |
| Margarine | Pork and beef mixes |
| Mayonnaise salad | Savoury pastries and biscuits |
| Pizza | Snacks and snack foods |
| Dairy-based desserts | Sweet pastries and biscuits |
| Ready-to-eat red meat dishes | Fresh vegetables and mushrooms |
| Sugar-sweetened beverages | Ready-to-eat poultry dishes and poultry patties |
| Ready-to-eat vegetable dishes | Baking products |
| Vegetable oils | Seasoning sauces |
| Whole milk | Alcohol beverages |
| Fresh potatoes | Fruits and berries |
| Sweeteners | Yoghurt |

| **Supplementary table 2**. The sum of annual carbon footprint (kg CO2-eq.) of and expenditure on total purchases of the participants (n=22,860) and the ratio of annual carbon footprint and expenditure. | | | | | |
| --- | --- | --- | --- | --- | --- |
|  | kg CO2-eq. /year | € / year | % of total CO2-eq. | % from total € | kg CO2-eq. / € |
| Beef and processed beef | 4 081 720 | 1 597 881 | 11.61 | 2.50 | 2.55 |
| Cheeses | 3 169 747 | 4 611 862 | 9.01 | 7.21 | 0.69 |
| Fresh vegetables and mushrooms | 2 263 960 | 4 307 422 | 6.44 | 6.73 | 0.53 |
| Uncategorized red and processed meat | 2 096 045 | 2 226 876 | 5.96 | 3.48 | 0.94 |
| Yoghurt | 2 033 395 | 3 056 162 | 5.78 | 4.78 | 0.67 |
| Pork and beef mixes | 1 941 484 | 916 182 | 5.52 | 1.43 | 2.12 |
| Pork and processed pork | 1 722 638 | 2 449 887 | 4.90 | 3.83 | 0.70 |
| Semi-skimmed milk and sour milk | 1 368 659 | 1 248 966 | 3.89 | 1.95 | 1.10 |
| Poultry and processed poultry | 1 321 791 | 2 641 021 | 3.76 | 4.13 | 0.50 |
| Fruits and berries | 1 284 869 | 4 052 495 | 3.65 | 6.33 | 0.32 |
| Skimmed milk and sour milk | 1 228 689 | 960 947 | 3.49 | 1.50 | 1.28 |
| Butter and butter-oil mixes | 1 102 059 | 942 657 | 3.13 | 1.47 | 1.17 |
| Coffee | 786 971 | 1 361 563 | 2.24 | 2.13 | 0.58 |
| Fish and seafood | 737 197 | 2 820 947 | 2.10 | 4.41 | 0.26 |
| Eggs | 650 763 | 729 884 | 1.85 | 1.14 | 0.89 |
| Creams | 640 947 | 729 907 | 1.82 | 1.14 | 0.88 |
| Sugar-sweetened beverages | 589 078 | 1 424 328 | 1.68 | 2.23 | 0.41 |
| Low-fiber bread | 517 515 | 2 313 391 | 1.47 | 3.62 | 0.22 |
| Sweets and coated nuts | 495 560 | 1 588 056 | 1.41 | 2.48 | 0.31 |
| Sweet pastries and biscuits | 484 195 | 1 869 653 | 1.38 | 2.92 | 0.26 |
| Ice cream | 473 572 | 1 160 295 | 1.35 | 1.81 | 0.41 |
| Alcohol beverages | 447 424 | 3 991 237 | 1.27 | 6.24 | 0.11 |
| High-fiber bread | 386 339 | 1 488 745 | 1.10 | 2.33 | 0.26 |
| Savoury pastries and biscuits | 375 668 | 929 119 | 1.07 | 1.45 | 0.40 |
| Fruit juices | 345 511 | 677 885 | 0.98 | 1.06 | 0.51 |
| Ready-to-eat red meat dishes | 315 807 | 678 461 | 0.90 | 1.06 | 0.47 |
| White rice and pasta | 298 463 | 530 234 | 0.85 | 0.83 | 0.56 |
| Snacks and snack foods | 280 749 | 1 162 965 | 0.80 | 1.82 | 0.24 |
| Chocolate and cocoa | 278 428 | 1 246 256 | 0.79 | 1.95 | 0.22 |
| Seasoning sauces | 237 614 | 614 581 | 0.68 | 0.96 | 0.39 |
| Desserts | 211 953 | 461 000 | 0.60 | 0.72 | 0.46 |
| Plant-based dairy alternatives | 207 039 | 853 556 | 0.59 | 1.33 | 0.24 |
| Pizza | 206 565 | 525 235 | 0.59 | 0.82 | 0.39 |
| Whole milk | 198 895 | 231 681 | 0.57 | 0.36 | 0.86 |
| Fresh potatoes | 193 191 | 456 306 | 0.55 | 0.71 | 0.42 |
| High-fiber cereal | 180 252 | 658 676 | 0.51 | 1.03 | 0.27 |
| Dairy-based desserts | 177 798 | 420 330 | 0.51 | 0.66 | 0.42 |
| Cooked and canned vegetables | 175 844 | 615 842 | 0.50 | 0.96 | 0.29 |
| Vegetable oils | 163 442 | 276 469 | 0.46 | 0.43 | 0.59 |
| Ready-to-eat poultry dishes | 142 051 | 325 653 | 0.40 | 0.51 | 0.44 |
| Peas, beans and lentils | 140738 | 417681 | 0.40 | 0.65 | 0.34 |
| Flour | 139 909 | 195 131 | 0.40 | 0.30 | 0.72 |
| Mayonnaise salad | 139 850 | 770 064 | 0.40 | 1.20 | 0.18 |
| Margarine | 121 419 | 392 085 | 0.35 | 0.61 | 0.31 |
| Frozen potato and potato trimmings | 106 812 | 385 707 | 0.30 | 0.60 | 0.28 |
| Baking products | 105 031 | 281 619 | 0.30 | 0.44 | 0.37 |
| Frozen vegetables | 104 161 | 368 567 | 0.30 | 0.58 | 0.28 |
| Ready-to-eat vegetable dishes | 83 953 | 235 525 | 0.24 | 0.37 | 0.36 |
| Nuts and almonds | 80 636 | 487 709 | 0.23 | 0.76 | 0.17 |
| Low-fiber cereal | 73 760 | 290 144 | 0.21 | 0.45 | 0.25 |
| Jam and marmalade | 71 677 | 269 508 | 0.20 | 0.42 | 0.27 |
| Dried fruits and berries | 59 891 | 281 062 | 0.17 | 0.44 | 0.21 |
| Ready-to-eat fish dishes | 54 510 | 169 936 | 0.16 | 0.27 | 0.32 |
| Canned and frozen fruits | 32 575 | 191 625 | 0.09 | 0.30 | 0.17 |
| High-fiber pasta and grain | 32 559 | 74 033 | 0.09 | 0.12 | 0.44 |
| Sweeteners | 335 | 16 356 | 0.00 | 0.03 | 0.02 |

**Supplementary figure 1.** Scree plot illustrating the eigenvalue of each principal component. The dashed line represents an eigenvalue of 1.
